# Factors Affecting Uptake of NaF-18 by the Normal Skeleton

**DOI:** 10.14740/jocmr1901w

**Published:** 2014-09-09

**Authors:** Aung Zaw Win, Carina Mari Aparici

**Affiliations:** a Department of Radiology, San Francisco VA Medical Center, 4150 Clement Street, San Francisco, CA 94121, USA; b Department of Radiology, University California San Francisco (UCSF), 500 Parnassus Ave, San Francisco, CA 94143, USA

**Keywords:** SUV, Bone, NaF-18 PET/CT, Bone scan

## Abstract

**Background:**

The primary aim of this study was to examine if factors such as renal function, height, weight and age could affect the uptake of sodium fluoride-18 (NaF-18) by the normal bone. This is the first study to examine the possible factors that can influence NaF-18 uptake in the normal bone.

**Methods:**

A retrospective study was done on NaF-18 PET/CT bone scans from January 2010 to May 2012 at our institution. All NaF-18 PET/CT studies used the same clinical protocol. Our excluding criteria were patients with abnormal renal function and patients with past history of cancer and metabolic bone diseases. Spearman’s correlation was used to analyze the data.

**Results:**

From our study (n = 11 patients), no correlation was found between SUV_max_ and serum creatinine and between SUV_max_ and age. However, significant correlations were found between SUV_max_ and height (cm) and between SUV_max_ and weight (kg) for thoracic 5, 7, 12 and lumbar 2 vertebral levels.

**Conclusion:**

Based on our findings, SUV_max_ values in NaF-18 PET/CT bone scans can vary depending on the patient’s height, weight and bone region. This information can be helpful in diagnosing and monitoring bone pathologies and can help explain the clinical findings.

## Introduction

SUV value is defined as the tissue concentration of tracer as measured by a PET scanner divided by the activity injected divided by body weight [1]. The uptake value is represented by pixel or voxel intensity value in the region of interest (ROI) of the image, which is then converted into the activity concentration. Previous studies on sodium fluoride-18 (NaF-18) PET/CT have reported correlations between SUV and lean body mass, and between SUV and total body weight [2-4]. However, SUV values vary depending on the organ of the body. SUV of bone depends on the blood flow to the area, exposed bone surface area, regional osteoblastic activity and on renal clearance [5]. The tracer uptake in bone does not plateau for several hours [1]. NaF-18 is excreted by the kidneys. Age-related changes of the bone are more pronounced in some locations compared to others. For example, parietal and occipital bones show more age-related changes compared to the rest of the skull [6].

The ability to reliably follow changes in SUV can be used to assess the changes due to therapy in cancer cases. It must be noted that blood flow varies among different bones [7]. The diffusion of NaF into the bone leads to a slow exchange of fluoride ions with hydroxide ions of the hydroxyapatite crystals, eventually forming fluoroapatite, a process that begins rapidly but takes many days to weeks to complete [5]. The rapid uptake of 18F-fluoride occurs preferentially at sites of high osteoblastic activity where bone remodeling is greatest. Hence the component of bone turnover being measured by 18F imaging is osteoblastic activity [8]. Yet, the tracer can accumulate in both osteoblastic and osteolytic lesions [9]. F18 ion has a high affinity for bone that leads to a large tissue to background ratio and hence good-quality images [10]. NaF-18 has two-fold greater tracer accumulation in skeletal system compared with Tc99m-MDP [11]. Unlike Tc99m tracer, there is no protein binding for NaF-18 and NaF-18 has faster blood and renal clearance [12]. NaF-18 PET/CT bone scan has less radiation exposure than Tc99m-MDP SPECT/CT [13]. Moreover, Tc99m-MDP tracer requires a delay of 2 h or greater before imaging can be performed [14]. The usual imaging time is 45 - 60 min for NaF-18 PET studies. Even-Sapir et al reported that NaF-18 PET/CT has a sensitivity and specificity of 100% respectively for detecting prostate cancer metastases [15]. It is certainly more sensitive and more specific than Tc99m-MDP bone scan.

NaF-18 PET/CT can detect skeletal metastases of tumors that typically have low FDG avidity, such as thyroid cancer or renal cell cancer [16]. A study by Nakai et al found that FDG PET had a low visualization rate in cases of osteoblastic bone metastases [17]. Moreover, NaF-18 can accurately differentiate between benign and malignant lesions [18]. No limitations to diet or physical activity are required for this exam, whereas, for FDG PET/CT, the patient has to fast overnight and limit physical activity to avoid increased FDG uptake by the muscles. For bone imaging, there is high uptake of FDG tracer by bone marrow, whereas with NaF-18, uptake of tracer by the bone marrow is negligible [7].

Increased NaF-18 accumulation is observed in inflammations, past trauma, fibrous dysplasia, Paget’s disease, hyperostosis frontalis, and myositis ossificans [11]. Quantitative 18F-fluoride PET may prove useful for the assessment of metabolic bone disorders such as renal osteodystrophy, osteoporosis, or Paget’s disease [19]. It has been used to assess metabolic, degenerative, traumatic, and neoplastic bone diseases. Many studies have been done on patients with osteoporosis and patients with bone metastasis. Cancer and metabolic bone diseases such as osteoporosis, osteopenia and Paget’s disease can alter the SUV values. Kurdziel et al carried out a prospective study with multiple myeloma and prostate cancer patients and they calculated SUV from NaF-18 PET/CT exams [5]. Research on SUV in NaF-18 exams is very limited. The primary aim of this study was to examine if factors such as renal function, height, weight and age could affect the uptake of sNaF-18 by the normal bone. This is the first study to examine the possible factors that can influence NaF-18 uptake in the normal bone.

## Methods

We retrospectively reviewed all the NaF-18 PET/CT bone scans done at our institution between January 1, 2010 and May 31, 2012. All NaF-18 PET/CT studies used the same clinical protocol. We excluded patients with histories of cancer and metabolic bone diseases such as osteopororsis, osteopenia and Paget’s disease. We included patients with degenerative changes in bone, patients who had arthroplastic surgery and patients with prosthetic devices in bone. We collected information on age, gender, serum creatinine values, height (cm), and weight (kg). The patients in this study (n = 11) had normal renal function based on the serum creatinine values. Two nuclear medicine physicians independently measured SUV_mean_ values in 31 locations (C1-C7, T1-T12, L1-L5, right and left femoral head, right and left humeral head, mid sternum on the level of T3, and right and left parietal bones) on the axial and appendicular skeletons. Areas with degenerative changes were avoided. The ROI used in this study was 826 mm^3^.

### Technique

Sixty minutes following the intravenous administration of NaF-18, CT transmission images without intravenous contrast were acquired from the vertex to the toes for attenuation correction and anatomic localization. This was followed by a PET scan over the same anatomical regions. A rotating 3D MIP, as well as axial, coronal and sagittal PET images with and without attenuation correction was interpreted. Acquired CT and PET/CT images were reviewed alongside the PET images. The dose of NaF-18 injected ranged from 9.5 to 12 mCi. Approximately 1 h after injection with the isotope, imaging was performed in a PET/CT scanner (GE STE 64 slice CT scanner, GE Healthcare, Waukesha, WI, USA) with a CT imaging system. CT scan was done for attenuation correction and anatomic localization. Imaging parameters were 140 kVp, 90 - 120 mA, 1.25 mm collimation with reconstruction as 3.75 mm thick sections by using a 512 × 512 matrix and a filtered back projection algorithm. Immediately after CT, PET images were obtained in two-dimensional mode for 4-min acquisitions at each level for one bed position. PET images were reconstructed by using a 128 × 128 matrix and an iterative-ordered subset expectation maximization algorithm.

### Statistics

We used the SPSS version 20 to analyze the data. Spearman’s correlation was used and a P value of less than 0.05 was considered significant for this study.

## Results

The characteristics of patients are shown in Table 1. The average normal SUV_max_ values from 11 patients were cervical vertebrae 6.84, thoracic vertebrae 7.36, lumbar vertebrae 7.27, femoral head 2.22, humeral head 1.82, mid sternum 5.51, and parietal bone 1.71. The mean age of the patients was 64.8 years and ranged from 42 to 89. The serum creatinine levels ranged from 0.64 to 1.29 (normal 0.6 - 1.3). The mean height was 174.79 cm (range 157.5 - 185.4) and the mean body weight was 83.27 kg (range 65 - 108). The dose of NaF-18 injected ranged from 9.5 to 12.6 mCi depending on the weight of the patient. We found no significant correlation between SUV and serum creatinine. There were also no significant correlations between SUV and age. However, significant correlations were found between SUV_max_ and height (cm) and between SUV_max_ and weight (kg) for thoracic 5, 7, 12 and lumbar 2 vertebral levels.

**Table 1:** Patient Characteristics

Patient	Age	Reason for NaF-18 PET/CT bone scan
1	54	Abnormal right femur, to rule out metastasis
2	50	Assess right seventh rib incidental finding
3	66	Evaluate for lytic lesions found on CT
4	62	Presumed metastasis to bones
5	89	Sclerotic lesion on X-ray
6	42	To rule out skeletal coccidioidomycosis
7	63	Status post left total hip arthroplasty with bone pain
8	65	Knee joint replacement
9	62	Arthropathy of left ankle
10	83	Benign prostatic hyperplasia (BPH)
11	77	Elevated PSA

**Figure 1:**
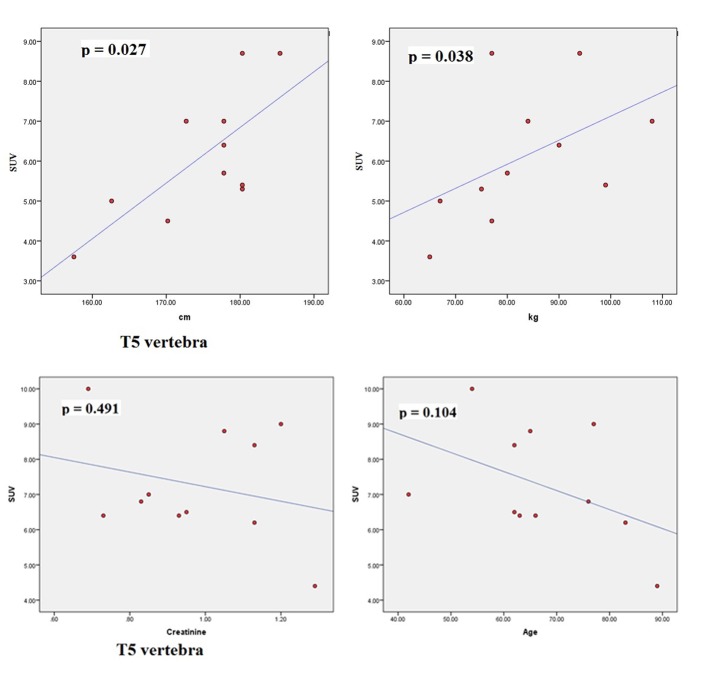
Scatter plots correlating SUV_max_ of T5 with height (cm), weight (kg), creatinine, and age (years).

**Figure 2:**
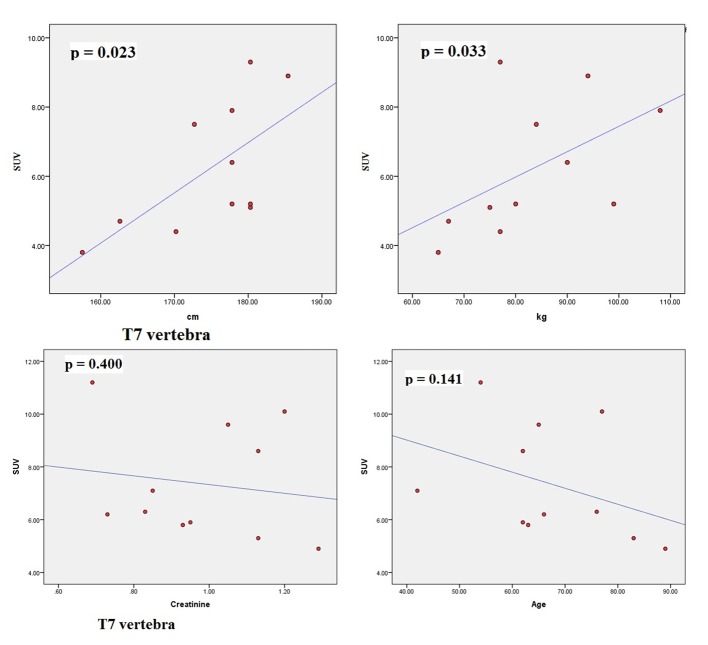
Scatter plots correlating SUV_max_ of T7 with height (cm), weight (kg), creatinine, and age (years).

**Figure 3:**
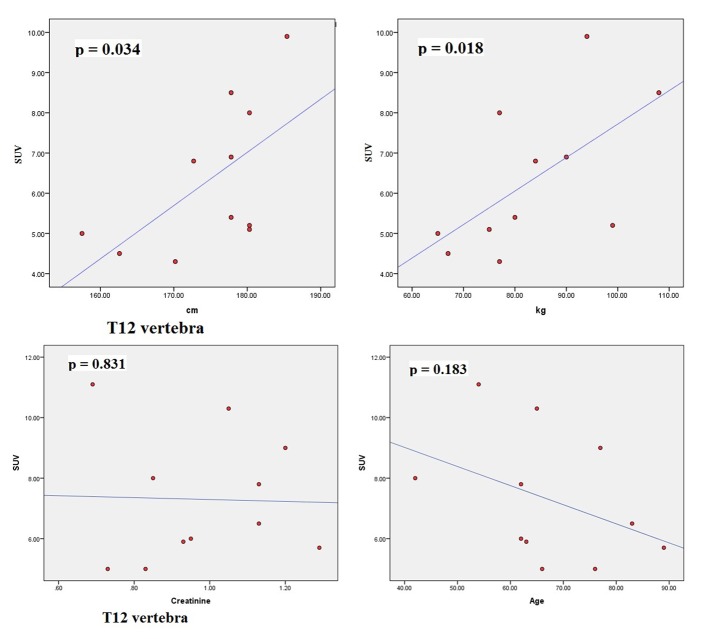
Scatter plots correlating SUV_max_ of T12 with height (cm), weight (kg), creatinine, and age (years).

**Figure 4:**
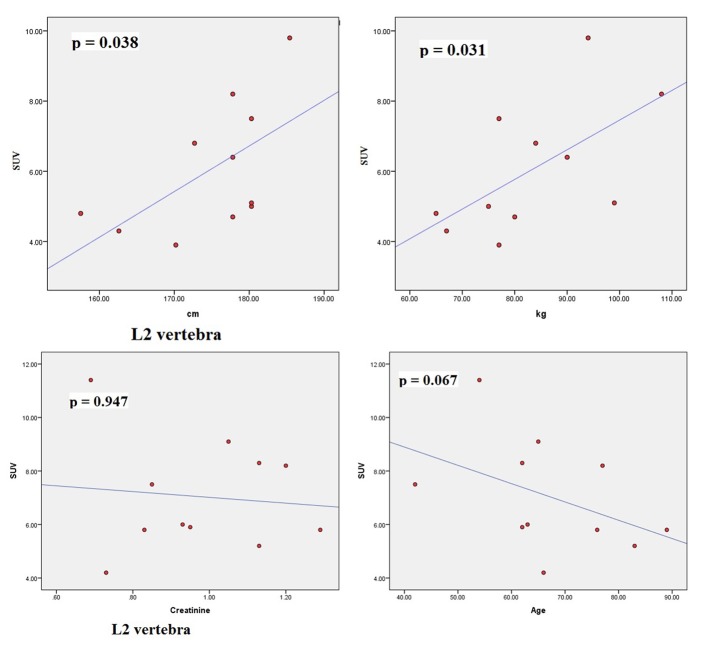
Scatter plots correlating SUV_max_ of L2 with height (cm), weight (kg), creatinine, and age (years).

The scatter plots of linear regression lines are shown in Figures 1-4.

## Discussion

NaF-18 tracer is eliminated from the body by the kidneys. However, we did not find a significant correlation between SUV and serum creatinine. One possible explanation may be that we would start to see a significant correlation once the serum creatinine value is above the normal range. All of our patients had serum creatinine values within the normal range and within that range, changes in SUV due to changes in creatinine level may be minimal. Another reason may be that nine out 11 (82%) patients in this study were overweight or obese. Creatinine production rate is decreased with reduced lean body mass [20]. Creatinine is formed in muscle and it is proportional to muscle mass. Older patients have reduced muscle mass. Moreover, commonly used drugs such as aspirin can have an effect on creatinine levels. From our study, age does not seem to affect the SUV uptake by bone. The mean age of our patients with past histories of arthralgia, joint replacement and osteoarthritis was 58.8 years, compared to the mean age of 64.8 years for the whole study population. As a result SUV uptake in younger patients may be high compared to older patients in this study. This might contribute to the fact that we found no significant correlation between age and SUV.

Osteoblastic activity decreases with age. So, it is not surprising that we observed a negative trend for age in all four bone levels shown in the graphs. Serum creatinine reflects kidney function and glomerular filtration rate (GFR). Renal fluoride clearance is related to the GFR, and NaF-18 PET/CT was used to assess renal function in rats [21]. Fluoride ions are freely filtered in the glomeruli but they also undergo tubular reabsorption [22]. Tubular reabsorption of fluoride increases with decreasing GFR [22]. Therefore, a negative relationship is expected between fluoride ions (SUV) and creatinine. This agrees with our results. However, for both age and creatinine, we did not see a statistically significant association with SUV, even though a negative relationship existed.

There were significant associations between SUV_max_ and height (cm) and between SUV_max_ and weight (kg) for thoracic 5, 7, 12 and lumbar 2 vertebral levels. Increased weight puts mechanical stress on the spine. Mechanical stress enhances interleukin 11 expression and this stimulates osteoblast differentiation [23]. Increased osteoblastic activity is associated with high SUV uptake. It can be further assumed that in obese persons, there is a significant risk of osteoarthritis developing in thoracic and lumbar vertebra, particularly at T5, T7, T12 and L2 levels. Borenstein reported that the lumbar spine is a common location for osteoarthritis [24]. O’Neill et al studied 681 women and 499 men with osteoarthritis and they found that obesity (increased weight) was associated with osteoarthritis at L3 vertebra [25]. In our study, increased tracer uptake in L2 was associated with increased weight.

In the vertebral column, thoracic and especially the lumbar vertebra bear the majority of the body weight and they absorb the stress of lifting heavy objects [26]. As a result, lumbar spine has significant bone turnover [27] and this may be part of the reason why there was correlation between SUV of lumbar spine and height and weight. Suenaga et al measured the SUV of mandibular bone using NaF-18 PET/CT [28]. They find that by using this method, they can detect mechanobiological changes in bone due to bone turnover from wear. Another reason for the association between SUV and body weight in NaF-18 bone studies may be that weight causes changes in geometry and structure on certain bones in the body [26]. A correlation between SUV and total body weight was reported in FDG PET studies [2-4].

Keyes reported that PET images should be interpreted subjectively rather than quantitative interpretation of SUV [1]. In contrast, Cook et al reported that SUV has predictive value for evaluating bone metastases, whereas, qualitative (visual) interpretation tended to be less valuable [29]. SUV measurements from NaF-18 PET/CT have the potential to monitor treatment response [22]. In patients with osteoporosis, SUV obtained from NaF-18 PET/CT studies significantly decreased after treatment with bisphosphonates [30]. This shows that simple SUV measurements may be sufficient for monitoring disease response in metabolic bone diseases. Simplified SUV uptake measurements may be suitable substitutes for more complex kinetic modeling [22].

Cancellous bone is less dense than cortical bone. It typically occupies the interior region of bones, and is highly vascular [3]. Thus, cancellous bone can have more SUV uptake than cortical bone. Cortical bone forms most of the bone mass, but it represents the minority of bone surface [22]. By comparison, cancellous bone forms only 20% of the bone mass but accounts for 80% of the bone turnover associated with remodeling [22]. The spine is rich in cancellous bone and it is the best site for quantitative assessment of bone metabolism because bone turnover is greater than that observed at other skeletal sites [31]. Lastly, unlike the prospective study of NaF-18 by Kurdziel et al, this is a retrospective study and the dosage of NaF-18 in our studies was the same as that used in routine exams [5]. Thus, our results are comparable to other routine exams.

### Conclusion

Many factors such as age, lean body mass, height, gender, and weight can possibly influence SUV values in NaF PET/CT imaging. Based on our findings, SUV_max_ values in NaF-18 PET/CT bone scans can vary depending on the patient’s height, weight and bone region. NaF-18 tracer is eliminated from the body by the kidneys; however, we did not find a significant correlation between SUV and serum creatinine. Simplified measurements of SUV on NaF-18 PET/CT promise to improve clinical application. This information can be helpful in diagnosing and monitoring bone pathologies and can help explain the clinical findings. Further studies involving multiple institutions and larger sample sizes can be done to further confirm our findings.
